# Impacts of Curcumin Treatment on Experimental Sepsis: A Systematic Review

**DOI:** 10.1155/2023/2252213

**Published:** 2023-01-30

**Authors:** Barbara Martins Vieira, Marcos Antonio Ferreira Caetano, Mara Taís de Carvalho, Felipe dos Santos Arruda, Fernanda Dias Tomé, Jordana Fernandes de Oliveira, Danilo Figueiredo Soave, Jonathas Xavier Pereira, Mara Rubia Nunes Celes

**Affiliations:** ^1^Department of Bioscience and Technology, Institute of Tropical Pathology and Public Health, Federal University of Goias, Goiania, Goias, Brazil; ^2^Department of Anatomy, Institute of Biomedical Science, University of São Paulo, São Paulo, Brazil; ^3^Department of Animal Science, School of Veterinary and Animal Science, Federal University of Goiás, Goiania, Goias, Brazil; ^4^Department of Morphofunctional, Faculty of Medicine of Goianesia, University of Rio Verde, Goianesia, Goias, Brazil

## Abstract

**Background and Aims:**

Sepsis is defined as a life-threatening organ dysfunction due to a dysregulated host immune response to an infection. Curcumin is a yellow polyphenol derived from the rhizome of Curcuma longa with anti-inflammatory and antioxidant properties scientifically proven, a condition that allowed its use as a tool in the treatment of sepsis. Thus, the purpose of this article was to systematically review the evidence on the impact of curcumin's anti-inflammatory effect on experimental sepsis.

**Methods:**

For this, the PubMed, MEDLINE, EMBASE, Scopus, Web of Science, and LILACS databases were used, and the research was not limited to a specific publication period. Only original articles in English using *in vivo* experimental models (rats or mice) of sepsis induction performed by administration of lipopolysaccharide (LPS) or cecal ligation and perforation surgery (CLP) were included in the study. Studies using curcumin in dry extract or with a high degree of purity were included. At initial screening, 546 articles were selected, and of these, 223 were eligible for primary evaluation. Finally, 12 articles with full text met all inclusion criteria. Our results showed that curcumin may inhibit sepsis-induced complications such as brain, heart, liver, lungs, and kidney damage. Curcumin can inhibit inflammatory factors, prevent oxidative stress, and regulate immune responses in sepsis. Additionally, curcumin increased significantly the survival rates after experimental sepsis in several studies. The modulation of the immune response and mortality by curcumin reinforces its protective effect on sepsis and indicates a potential therapeutic tool for the treatment of sepsis.

## 1. Introduction

According to the Third International Consensus Definitions for Sepsis and Septic Shock (Sepsis-3), sepsis is defined as life-threatening organ dysfunction due to a host's unregulated immune response to infection [1–3]. It is characterized by a set of hemostatic, biochemical, immunological, and metabolic changes that can lead to multiple organ dysfunctions and progress to septic shock (a subset of sepsis with critical circulatory, cellular, and metabolic abnormalities that increase the risk of mortality) and even death [1]. It is associated with high mortality rates, and the pathogenesis remains unclear despite numerous medical advances, making it difficult to develop new therapies and effective approaches [4]. Sepsis has a unique and time-sensitive clinical evolution, and its incidence has been increasing every year [5]. In 2017, the estimate of incident cases of sepsis worldwide was 48.9 million, with 11.0 million sepsis-related deaths reported, which represents about 19.7% of all global deaths [6]. In addition to the increasing incidence and deaths related to sepsis worldwide, its management is very expensive for health services, costing about $24 billion annually for the USA [7, 8]. For this reason, sepsis is considered a serious global challenge [9].

Sepsis develops by exacerbating the inflammatory response and suppressing immunity, with organ dysfunction being a key factor in its development [4, 10]. Its pathophysiology seems to be involved with a series of disorders in the organism such as dysregulation of the immune system, hemostasis, immunosuppression, cellular, tissue, and organ dysfunction [11, 12].

Despite scientific and technological advances aimed at the development of new drugs to combat sepsis, mortality rates remain high [13]. From this perspective, the search for new natural or synthetic compounds/drugs with anti-inflammatory effects against sepsis is of great importance. Curcumin is a yellow polyphenol derived from the rhizome of Curcuma longa with anti-inflammatory, antioxidant, and immunomodulatory activities [14, 15], frequently studied in the treatment of endocrine, respiratory, and liver disorders [16]. This compound has also been described for the treatment of several inflammatory diseases, such as inflammatory bowel disease [16] and rheumatoid arthritis [17].

Taking into account the understanding effects of curcumin in the treatment of sepsis and the fact that few systematic reviews are showing the anti-inflammatory effect of curcumin, the purpose of this study was to conduct a systematic review of studies in animals using the cecal ligation and puncture (CLP) sepsis induction models (considered the gold standard) and the endotoxemia model, induced by intraperitoneal injection of lipopolysaccharide (LPS) to describe the anti-inflammatory effects of curcumin on sepsis and its possible mechanism of action.

## 2. Methodology

### 2.1. Protocol and Registration

This systematic review was carried out according to the Preferred Reporting Items for Systematic Reviews and Meta-Analysis checklist [18] under the protocol number CDR42019146945 in the International Prospective Register of Systematic Reviews (https://www.crd.york.ac.uk/prospero/display_record.php?RecordID=146945). Additionally, as supplementary material (available here), we present the 27-item PRISMA 2020 checklist, used to design and report a robust protocol for this systematic review.

### 2.2. Eligibility Criteria

#### 2.2.1. Inclusion Criteria

For this review, the minimum degree to be included (in accordance with PRISMA 2020) in the text was only articles in English available in full in scientific databases (PubMed, MEDLINE, EMBASE, Scopus, Web of Science, and LILACS). *In vivo* experiments (rats or mice) with a sepsis model induced by LPS administration or CLP were included using a dried extract of curcumin or with a high degree of purity.

#### 2.2.2. Exclusion Criteria

Studies in which the text was not available in full form or published in a language other than English were discarded. Additionally, experiments performed in vivo, but not in rats or mice, using a “two-hit” bacterial bolus-induced sepsis model or any other model were also rejected. In vitro studies, review articles, conference proceedings or abstracts, and dissertations were also not accepted.

### 2.3. Research Strategies

Search strategies were carried out by three pairs of researchers (PR) in the following electronic bibliographic databases: PubMed (public full-text archive of biomedical and life sciences journal literature), MEDLINE (National Library of Medicine (NLM) journal citation database), EMBASE (medical literature database), Scopus (Elsevier's database with a broad representation of scientific production in Latin America), Web of Science (independent global citation database), and LILACS (Latin American and Caribbean Literature in Health Sciences database). The search strategy used was “sepsis OR septic shock AND curcumin” (MeSH term - Medical Subject Headings to index citations). The research included all articles published on or before July 8, 2019, with no publication time restrictions.

In the first phase, the three pairs of researchers (PR1: MAFF and BMV; PR2: MTC and JFO; and PR3: FSA and FDT) performed the initial analysis based on the titles and abstracts of the manuscripts. Articles that did not meet the minimum inclusion criteria were excluded, and those that did were included in the second phase. In the case of disagreement, a third researcher (MRNC) intervened. In the second phase, the articles were independently read by two researchers (MAFF and BMV) to select acceptable articles in agreement with the inclusion criteria. Articles that did not fit the inclusion criteria were excluded, and each article's reason for exclusion was recorded. The third researcher (MRNC) intervened in any disagreement.

### 2.4. Data Collection

One researcher (MAFF) collected the necessary information from the articles selected for the study, and a second researcher (MTC) checked the data extracted by the first author. Any disagreements were resolved by discussion and, where necessary, a third researcher (MRNC) was involved.

### 2.5. Risk of Bias Analysis

The quality of information and the risk of studies were systematically performed using the SYRCLE tool (SYRCLE's risk of bias) for in vivo studies [19]. Two researchers (MAFF and MTC) classify each item judged as “yes,” “no,” or “uncertain” for each article. When there were discrepancies, a third researcher (MRNC) made the final decision.

## 3. Results

### 3.1. Study Selection

**Figure 1:**
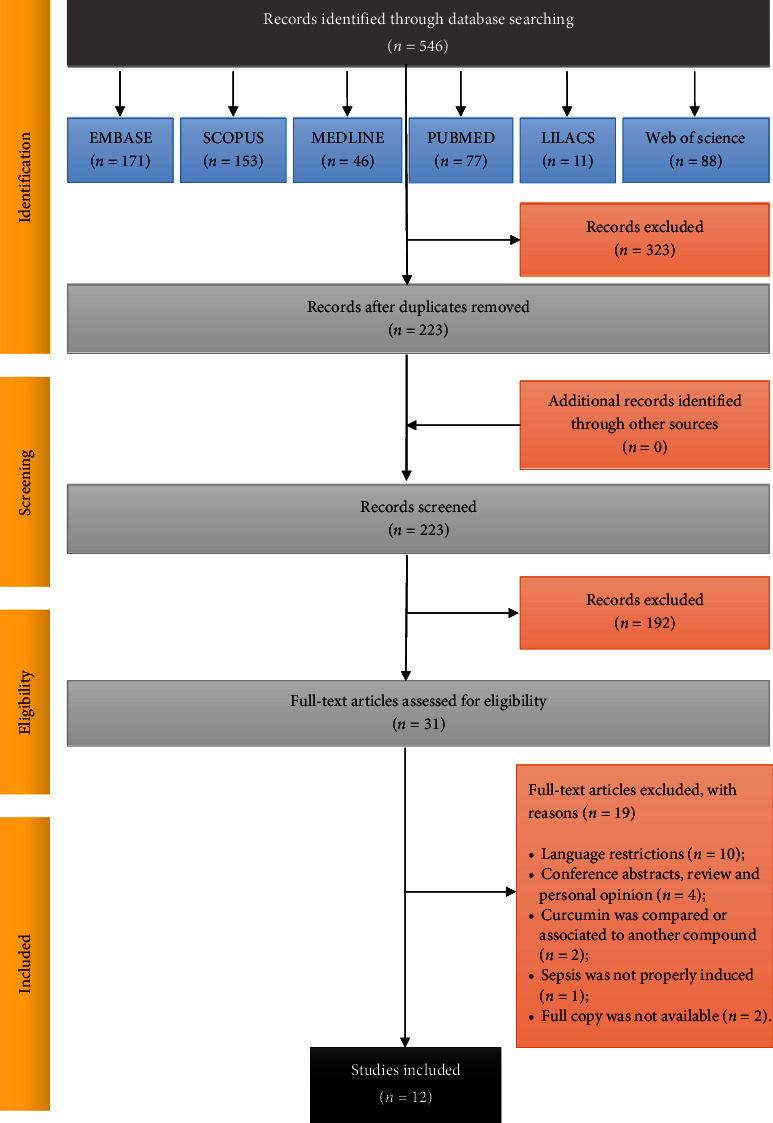
Flow diagram of literature search, screening, and selection process adapted from PRISMA [18].

A total of 546 articles were selected individually from different electronic databases. After removing duplicate studies, 223 articles remained for analysis of titles and abstracts. After screening, 192 articles were excluded for not meeting the predefined inclusion criteria (for example, not being available in full text, not written in English, not administering curcumin in the form of dry extract, or with the same level of purity), and 31 articles remained. The remaining 31 studies were submitted for evaluation, and 19 articles were excluded after a critical analysis of the title, abstracts, and full texts. Finally, only 12 articles were selected (Figure 1).

### 3.2. Characteristics of the Studies

The studies were carried out in four different countries: China (66.67%, *n* = 8) [20–27], Turkey (*n* = 2) [28, 29], India (*n* = 1) [30], and the United States (*n* = 1) [31]. The articles were published between 2009 and 2019. All articles correspond to in vivo studies.  Table 1 shows the characterization of the different studies included in this systematic review.

Different studies evaluated the effects of curcumin in animals (rats or mice) subjected to sepsis induced by CLP (66.67%, *n* = 8) [21, 22, 24–27, 29, 31] or by LPS administration (33.33%, *n* = 4) [20, 23, 28, 30]. Most studies used 3 (41.66%, *n* = 5) [20, 22, 25, 29, 31], 5 (25%, *n* = 3) [23, 24, 27], 4 (16.66%, *n* = 2) [26, 28], or 6 (16.66%, *n* = 2) [21, 30] experimental groups, respectively, with the number of animals per group ranging from five to sixty [20–31].

Curcumin was diluted in DMSO in 33.33% of studies (*n* = 4) [22, 24, 27, 30], in saline in 33.33% (*n* = 4) [25, 26, 28, 31], in corn oil in 8.33% (*n* = 1) [21], or in carboxymethylcellulose in 8.33% (*n* = 1) of studies [20], and in two of the studies (16.67%) the vehicles used were not reported [23, 29].

Curcumin was administered by intraperitoneal injection in seven studies (58.33%) [22, 24, 25, 27, 29–31] and by gavage in five studies (41.67%) [20, 21, 23, 26, 28] at concentrations of 10 mg/kg [30], 20 mg/kg [20, 30], 50 mg/kg [21, 26, 27], 80 mg/kg [23], 100 mg/kg [20, 22, 26, 31], 200 mg/kg [21, 24, 25], and 1200 mg/kg [28]. Only two studies administered curcumin before induction of experimental sepsis [30, 31], both in a single dose. The routes of administration were intraperitoneal (100 mg/kg) [31] or a combination of two routes at different doses, intraperitoneal (20 mg/kg) and intranasal administration (10 mg/kg) [30]. In nine studies, curcumin was administered after sepsis induction using multiple applications ranging from 2 to 45 [20–23, 25–29]. Only one study did not report the time of administration of doses (before or after the induction of sepsis); it just reported that two applications of 200 mg/kg were performed [24]. In addition, four studies tested different doses of curcumin in different experimental groups, testing 50 mg/kg, 100 mg/kg, and 200 mg/kg [21], or 50 mg/kg and 100 mg/kg [26], or 50 mg/kg and 200 mg/kg [27].

To assess the effects of curcumin on sepsis, the different studies collected blood, serum, or plasma (83.33%, *n* = 10) [20, 22, 23, 25–27, 29–31] in addition to organs such as the liver (41.67%, *n* = 5) [20, 23, 26, 28, 29] lung (33.33%, *n* = 4) [21, 24, 27, 30], kidney (33.33%, *n* = 4) [20, 21, 28, 29], brain (16.67%, *n* = 2) [22, 31] spleen (8.33%, *n* = 1) [21] heart (8.33%, *n* = 1) [25], and intestine (8.33%, *n* = 1) [28].

### 3.3. Summary of Results

The protective effect of curcumin on the survival rate of septic animals has been demonstrated in the studies. Survival rates were analyzed using the Kaplan-Meier survival curve and compared using the log-rank test [21, 22, 26, 27, 29]. Pretreatment of mice with curcumin before induction of sepsis showed significant improvement in survival compared to untreated septic mice [30, 31]. Mice treated with curcumin after sepsis induction also showed improved survival. Curcumin improved the survival rate of rats with CLP-induced acute lung injury (ALI) by 40%-50 [27]. The survival rate was significantly improved by up to 60% in curcumin-treated mice compared to 20% in untreated mice [21].

Improvement in survival was also observed when different concentrations of curcumin were used. Septic rats treated with curcumin showed increased survival rates, approximately 80% when treated with 50 mg/kg curcumin and 90% when treated with 200 mg/kg curcumin compared to septic untreated animals, which showed 40% of survival, suggesting that the survival rate may be related to the administered dose [22]. Furthermore, animals treated with curcumin before LPS injection had reduced lethality, an improvement directly related to curcumin dosage [26].

In summary, the analysis of the studies shows that curcumin acts as an anti-inflammatory by inhibiting reactive oxygen species generation via inhibiting oxidative stress [21, 26, 28, 30], regulating cytokine production as a result of which it blocks the oxidation process reducing inflammation [21, 26, 29, 30], and reducing inflammatory cells infiltration at different organs and tissues [26–28, 30]. Curcumin promotes the reduction of inflammation in several organs, such as the lungs, liver, kidneys, brain, heart, spleen, and intestine [20–31], which directly impacts improved survival, reaching levels of 90% in the curcumin-treated groups and 40% in the control groups [27].

Pulmonary findings showed that curcumin inhibited the production of inflammatory cytokines [27, 30], expression of proteins associated with oxidative stress [23], and alveolar exudation and modulation of platelet adhesion leading to a reduction in degeneration and cell death by necrosis [24, 29], consequently reducing the pulmonary edema and injury, in addition to promoting an improvement in survival between 40 and 50% [27]. In the liver, curcumin promoted the reduction of inflammatory processes through the reduction of cytokine production [20, 28] and decreased expression of proteins associated with oxidative stress and normalization of liver enzyme levels, such as alkaline phosphatase, aspartate aminotransferase, and alanine aminotransferase [20, 23]; in addition, decreased cell degeneration and necrosis [28] with reduced damage to hepatocytes were related [20, 29]. Reduction of histological damage with the improvement of inflammatory lesions and reduction of degeneration and necrosis of glomeruli and renal tubules were observed in curcumin-treated septic animals [20, 21, 28, 29]. Protective effects of curcumin were observed on cardiac function, with improvement in contractility by increasing ejection fraction and fractional shortening in septic rats [25]. Additionally, curcumin alleviates myocardial inflammation and reduces the structural damage of cardiomyocyte cells in sepsis [25]. Moreover, curcumin improved the blood-brain barrier integrity by attenuating brain edema, decreasing apoptosis, and reducing mitochondrial dysfunction in septic mice [22, 31].

### 3.4. Risk of Bias Analysis

All 12 studies [20–31] included were considered with unclear risk of bias according to SYRCLE's risk of bias analysis (Table 2). Information about randomization, allocation, and blinding process was not clearly described in articles, which are required for SYRCLE's assessment.

## 4. Discussion

### 4.1. Curcumin on Animal Survival Rates

Despite significant advances in the treatment of critically ill patients, sepsis remains associated with high mortality, and most survivors present long-term cognitive dysfunction [32]. The lack of data limits the prediction of global cases; however, there are estimates of about 5.3 million deaths per year [33]. In 2020, Fleischmann-Struzek et al. [34] performed a meta-analysis study with data from 22 countries. In this study, they adverted the high incidence of sepsis in all studied regions and reported that one in four patients with sepsis did not survive hospitalization [34].

During the literature search, we reviewed several articles that explain the high therapeutic value and very low toxicity of curcumin in different diseases. A significant improvement in survival rate was observed in animals treated with curcumin before and after sepsis induction by the CLP or LPS model [21, 27]. This enhancement of survival was also reported when the studies used different concentrations of curcumin showing survival rates of 80% (50 mg/kg) and 90% (200 mg/kg), whereas control rat groups had a 40% survival rate, showing the efficiency of curcumin in different doses [21, 23, 27].

Although curcumin has demonstrated potential therapeutic in sepsis through the improvement of survival rate due to the inhibition of the inflammatory mediators, oxidation processes, and oxidative stress [23, 26, 30, 35–41] and reduction of tissue injuries [23, 24, 26, 28–30, 42, 43] and has presented no severe toxicity on animals and humans [20–29, 31], more studies about their therapeutic effect need to be performed.

If we look at sepsis and septic shock from the perspective of a critically ill patient, there are a few questions to be asked: What is the best route for curcumin administration? How many doses are necessary to induce protection? How could maintaining curcumin's chemical and physical properties increase its bioavailability in the organism? All of these questions are interrelated, and the answers will be more easily addressed if we continue to understand the basic mechanisms implicated.

### 4.2. Curcumin: Anti-inflammatory Effects and Tissue Damage Reduction

For centuries, curcumin has been used for its anti-inflammatory properties and, in recent decades, it has become the target of many studies that have tried to elucidate its anti-inflammatory mechanism of action. In animal models, curcumin prevents the formation of ethanol-induced liver damage [41] as well as experimental alcoholic and nonalcoholic pancreatitis [36]. According to Gaddipati et al. [37], pretreatment of animals with curcumin attenuates the increased production of proinflammatory cytokines induced by hemorrhage and inhibits the activation of nuclear factor B (NF-*κ*B) and activated protein-1 (AP-1), shown to subdue inflammation by different mechanisms. Curcumin led to a significant reduction in TNF-*α* and IL-8 in bronchoalveolar lavage and a reduction in TNF-*α* in serum [21, 26, 30]. In addition, iNOS mRNA was suppressed with the use of curcumin [20]. Serum levels of IL-1*β* and IL-6 also significantly decreased with the use of curcumin in the treatment of sepsis, while IL-10 showed increased plasma levels [27, 28]. Treatment with curcumin led to the regulation of protein expression of IKK*β*, I*κ*B*α*, p-NF-*κ*B, TNF-*α*, IL-1*β*, and IL-18 stimulated by sepsis [23]. Transforming growth factor-*β*1 gene expression significantly decreased with treatment, and its plasma levels were also regulated with the use of curcumin [24, 30]. Curcumin has a cytoprotective action and is also effective in suppressing the hepatic microvascular inflammatory response in endotoxemia, as demonstrated by its inhibitory effects on Kupffer cell activation, neutrophil adhesion, and endothelial cell edema [38–40].

Curcumin can be useful in the therapy of organ dysfunction associated with sepsis, shock, and other diseases related to local or systemic inflammation [28]. Analyzed studies show that curcumin promoted neuroprotective properties in a clinically relevant model of sepsis [22]; significantly reduced the proportion of wet/dry lung weight in acute lung injury induced by sepsis in rats [27]; significantly reduced the total cell count, neutrophil infiltration, and lymphocyte count after the administration of curcumin for 45 days [26]; and significantly reduced the number of inflammatory cells in the bronchoalveolar fluid after intraperitoneal administration of 20 mg/kg in the LPS sepsis model [30].

Among the histopathological benefits of curcumin is the attenuation of hydropic degeneration of hepatocytes, reduction of necrosis in the liver lobes, and diminution of inflammatory infiltration in the portal areas [28]. Dilation and congestion of hepatic sinusoids [29] were reduced and associated with improved collagen deposition and reduced fibrosis [30]. In lung tissue, curcumin treatment reduced fibrin exudation in the alveolar space and cell necrosis, as well as resulted in the absence of red cell exudation/hemorrhage [21, 26, 43], decreased inflammation, bronchoconstriction, and mucus secretion in the airways, and improved collagen deposition induced by LPS [30]. Considered a vital organ during the sepsis infection, the disordered progression of the disease could culminate in acute lung injury and acute respiratory distress syndrome (ARDS) [30], a common cause of respiratory failure in critically ill patients.

In kidneys, a reduction of glomerular and tubular damage [29] attenuation of acute tubular epithelial necrosis [20, 23], a reduction in inflammatory cell infiltration [29], and an improvement in tubular dilation and inflammatory infiltrate [21] were reported. Chen et al. [21] suggested that the protective effects of curcumin are associated with the modulation of the NF-*κ*B signaling pathway and consequent reduction in the expression of IL-1*β*, IL-6, IL-8, and TNF-*α* that prevent the formation of kidney inflammatory lesions. Curcumin also prevented renal tubular oxidative damage by reducing ROS production. Furthermore, curcumin restored mitochondrial homeostasis by regulating OPA1, modulating DRP1 expression, and inhibiting caspase-3 activation [44]. Together, all results show that even with incomplete knowledge of the mechanism of action of curcumin for the modulation of inflammation, we can predict its beneficial effects on the outcome of mortality in sepsis.

### 4.3. Curcumin Effects on Molecular and Biochemical Parameters

Kothari et al. [45] demonstrated that during sepsis, the exacerbated activation of neutrophils in peripheral blood and tissues increases the myeloperoxidase (MPO) release into both the phagolysosomal compartment and the extracellular environment, leading to a strong oxidative activity of MPO. However, curcumin also exhibits antioxidant properties upon MPO activity. Different studies showed that MPO activity was blocked by the curcumin treatment in the lung [26], liver [23], and hepatic and renal tissues [29]. Additionally, malondialdehyde (MDA) was also significantly attenuated by treatment with curcumin in the lung [30] and liver and kidney tissues [29], and a decrease in MDA was also observed in the plasma [28]. Doses of 50 and 100 mg/kg of curcumin also significantly reduced lipid peroxidation by 25% and 39.28%, respectively [26]. With the administration of curcumin, superoxide dismutase (SOD) activity increased or there was a small reduction in its plasma levels [23]. The improvement in the liver SOD activity was observed with different concentrations of curcumin (20, 40, and 80 mg/kg) [27], and a significant increase in SOD activity was observed with 50 and 100 mg/kg curcumin treatments [26].

The administration of curcumin normalizes the mRNA expression of the PI3K/AKT signaling pathway, which increases with sepsis induction and is related to the progression of apoptosis [23]. Additionally, the suppression of other proteins that contribute to the onset of apoptosis (Bad, Bcl-xL, Cyto-c, Apaf1, and cleaved Caspase-3/6/9) was observed, in association with the inhibited expression of mRNA related to LPS-induced apoptosis in the liver [23, 41]. Na+/K+-ATPase activity increased significantly with the use of curcumin [29]. Its use also reduced hepatic and serum levels of ALT, AST, and ALP, related to liver injury, with significant differences between doses [10, 23]. Restoration of CAT activity with treatment and reduction of O_2_^−^, H_2_O_2_, and NO were also observed [23]. Curcumin significantly increased catalase activity at doses of 50 and 100 mg/kg [26], reduced F4/80+ CD11c+ cells in the spleen of rats [20], and, in addition, decreased the expression of FOXP3 and the proliferation activity of CD4+ CD25+ splenic Treg cells in a dose-dependent manner in septic mice [21]. Curcumin in sepsis reduced cerebral mitochondrial dysfunction (MMP, ROS, and mitochondrial complex activity I), apoptosis in neurons, and expression of BAX and increased the expression of BCL-2 [22]. P-selectin expression was reduced with the use of curcumin in the brain, liver, and kidney tissues [10]. Glutathione (GSH) was elevated in the kidney [29] and liver [23, 29] in animals treated with curcumin, and GSH-px was also recovered [23]. Cardiac troponin I (cTnI), which increases after sepsis induction, is significantly lower with the administration of curcumin [25].

Herein, the results revealed that in sepsis, treatment with curcumin, a bioactive compound with proven immunoregulatory and antioxidant properties, has contributed to the growing interest in understanding its effects, especially its anti-inflammatory action, with decreased release of inflammatory mediators and consequent reduction of damage during sepsis and also of its antioxidant effect by regulating the production of free radicals and increasing levels of antioxidant enzymes at different doses [21, 46, 47].

In addition, several experimental studies use different curcumin concentrations, forms of administration, and treatment periods [48–50]. According to the American regulatory agency Food and Drug Administration (FDA), its intake is also considered safe for humans, even in larger amounts, for example, in the Indian diet (daily dose of 60 to 100 mg of curcumin) [51–54].

## 5. Limitations

The main limitations observed during the evaluation of the studies were related to the induction of sepsis, administration routes, doses, and administration intervals of curcumin.

The induction of sepsis using the cecal ligation and puncture (CLP) model can generate limitations because, according to Hubbard et al. [55], the gauge of the needle used to puncture the animals' intestine changes the intensity of the sepsis stimulus. Furthermore, the LPS concentrations used in the different studies were distinct. Together, these conditions could lead to different biochemical, molecular, and tissue responses in experimental sepsis.

Heterogeneous doses and different routes of administration for treatment with curcumin, lack of standardization for the intervals for the administration of curcumin, and also the lack of important information to assess the risk of bias in the selected articles were the important limitations found in carrying out this study.

## 6. Conclusions

Studies analyzed pointed to the positive effects of curcumin, demonstrating its ability to inhibit oxidative and inflammatory factors through the modulation of the immune response. However, further studies involving the mechanisms by which curcumin acts in the regulation and neutralization of antioxidant and inflammatory compounds in addition to tissue protection in several stages of the pathophysiology of sepsis and its complications are necessary to investigate its effect and possible mechanisms of action in humans.

## Figures and Tables

**Table 1:** Main characteristics of the included studies in the current review.

Author, year (ref)	Animal model	Experimental subject	Assays	Sepsis induction	Treatment	Results	Main conclusions
Memis et al., 2008 [28]	Rats Wistar albino (male)	Group I: control Group II: CUR (1.2 g/kg) Group III: LPS+placebo Group IV: LPS+CUR	Histopathological examination	LPS (1 mg in 500 *μ*L of sterile saline) i.p.	CUR (1.2 g/kg) through an orogastric tube, daily for 7 days after LPS administration	CUR attenuated hepatocellular hydropic degeneration, sinusoidal dilation, necrosis areas, and inflammatory infiltration in the liver; reduced inflammation, hyperemia, and mucosal ulceration in the small bowel; attenuated inflammation and necrosis of the proximal tubules in the kidney	CUR reduced organ dysfunction in rats with experimental sepsis

Vachharajani et al., 2010 [31]	Mice C57Bl/6 (male)	Group I: sham Group II: CLP Group III: CLP+CUR (100 mg/kg)	Intravital fluorescent video microscopy technique Evans blue (EB) leakage method Dual radiolabeling technique Survival rate	CLP	CUR (100 mg/kg) in saline i.p. pretreated with 48 h before CLP P<0.05	CUR significantly attenuated leukocyte and platelet adhesion in cerebral microcirculation, EB leakage in the brain tissue, and improved survival in mice with CLP. P-selectin expression in mice with CLP+CUR was significantly attenuated. CUR reduced platelet adhesion via modulation of the endothelium	CUR modulates leukocytes and platelet adhesion and blood-brain barrier (BBB) dysfunction in mice with CLP via P-selectin expression

Xiao et al., 2012 [27]	Rats Sprague-Dawley specific pathogen-free (male)	Group I: sham Group II: CLP Group III: CLP+DMSO Group IV: CLP+CUR (50 mg/kg) Group V: CLP+CUR (200 mg/kg)	Measurement of lung W/D ratio Measurement of lung injury in bronchoalveolar lavage fluid (BALF) Histopathological examination of the lung Measurement of MPO, MDA, and SOD activity Measurement of inflammatory cytokines Survival rate	CLP	CUR (50 mg/kg or 200 mg/kg) dissolved in 1% DMSO i.p. at 2 h and 12 h post-CLP	Treatment with CUR significantly attenuated the CLP-induced pulmonary edema and inflammation, as it significantly decreased lung W/D ratio, protein concentration, and the accumulation of the inflammatory cells in the BALF, as well as pulmonary myeloperoxidase (MPO) activity. CUR significantly increased superoxide dismutase (SOD) activity with a significant decrease in malondialdehyde (MDA) content in the lung. Caused downregulation of the inflammatory cytokines TNF-*α*, IL-8, and MIF levels in the lung, and CUR improved the survival rate of rats by 40%–50% with CLP-induced acute lung injury (ALI)	CUR protects against sepsis-induced acute lung injury in rats by reducing inflammatory cell infiltration, reactive oxygen species (ROS) generation, and regulating cytokine effects

Savcun et al., 2013 [29]	Rats of Wistar albino (both genders)	Group I: control Group II: CLP Group III: CLP+CUR (200 mg/kg)	Histopathological analyses Measurement of TNF-*α* and IL-1*β* levels Hepatic and renal tissues MDA and glutathione (GSH) levels MPO and Na+/K+-ATPase activities	CLP	CUR (200 mg/kg i.p.) in two equal doses just after surgery and at the 12-hour post-CLP	Serum TNF-*α* and IL-1*β* and tissue MDA and MPO values were higher, whereas tissue GSH and Na+/K+-ATPase values were lower, in the CLP group as compared to the sham group. These values in the CLP+CUR group were the inverse of those in the CLP group. As compared to the sham group, histopathological evaluation of the CLP group showed damaged hepatocytes, glomeruli, and tubules, whereas the damage was significantly reduced in the CLP+CUR group as compared to the CLP group	CUR has antioxidant and anti-inflammatory effects against the tissue damage likely caused by free oxygen radicals and lipid peroxidation induced by experimental sepsis in rats

Xu et al., 2013 [24]	Sprague-Dawley rats (male)	Group I: control Group II: sham Group III: CLP Group IV: DMSO Group V: CUR (200 mg/kg)	Lung wet/dry weight ratio Transmission electron microscopy Histopathological examination RNA isolation and analysis Real-time reverse transcription-polymerase chain reaction Enzyme-linked immunosorbent assay Protein determination Western blot analysis	CLP	CUR (200 mg/kg, 2 days, in DMSO, was administered i.p. 2 h and 12 h post-CLP	CUR treatment was found to significantly reduce lung wet/dry weight ratio in the sepsis-induced acute lung injury in rats at both 24 and 48 h. 24 h after the initial treatment, real-time PCR and Western blot analysis showed that the expression of TGF-*β*1 and SMAD3-dependent signaling pathway was significantly decreased in the CUR-treated group than other control groups (P b 0.05). In the CUR group, exudation of fibrin in the alveolar space and cell necrosis were less prominent, and there was no exudation of erythrocytes. CUR treatment prevented some sepsis-induced damage	CUR played a protective role in sepsis-induced ALI, possibly through the inhibition of the expression of the TGF-*β*1/SMAD3 pathway

Yang et al., 2013 [25]	Sprague-Dawley rats (male)	Group I: sham Group II: CLP Group III: CLP+CUR (200 mg/kg/d)	Determination of cardiac function by cardiac ultrasound, morphological changes of myocardial tissues and contents of cTnI, SOD, and MDA in plasma	CLP	CUR (200 mg/kg/d, 3 days) was administered i.p. in two equal doses just after the perforation and at twelve-hour postperforation	Treatment of rats with CUR significantly decreased the elevated cardiac troponin I (cTnI) levels and MDA in plasma and increased the levels of SOD after CLP. Moreover, CUR enhanced the myocardial contractility by increasing the decreased ejection fraction (EF) and fractional shortening (FS) in rats with sepsis-induced by CLP. In addition, CUR could alleviate the myocardial inflammation and structural damage of myocardial cells in sepsis induced by CLP	CUR has the protective effects on cardiac function in rats with sepsis

Zhao et al., 2016 [22]	Mice C57BL/6 (male)	Group I: control Group II: CLP Group III: CLP+CUR (100 mg/kg)	Evaluation of survival rate BBB permeability TUNEL-apoptosis kit for staining mitochondria and cytosolic fraction isolation Mitochondrial membrane potential (MMP) Measurement of mitochondrial ROS production Mitochondrial complex I activity measurement Western blot	CLP	CUR (100 mg/kg) dissolved in 1% DMSO (in normal saline) was administrated i.p. of each time, at 3-, 12-, and 24-hour post-CLP	CUR improved survival rate, attenuates brain edema, enhanced BBB integrity, decreased apoptosis, and attenuated mitochondrial dysfunction in septic mice	CUR improved the survival of mice with sepsis and ameliorated brain injury

Zhong et al., 2016 [23]	Mice C57BL/6 (male)	Group I: control Group II: LPS Group III: LPS+CUR (20 mg/kg) Group IV: LPS+CUR (40 mg/kg) Group V: LPS + CUR (80 mg/kg)	Biochemical hepatic function examination Measurement of liver O_2_^−^ and H_2_O_2_ levels and intracellular ROS production Measurement of TNF-*α*, IL-1*β* and IL-18 levels Liver histological analysis Semi-quantitative RT-PCR analysis Western blot	LPS (5 mg/kg) i.p.	CUR (20, 40, and 80 mg/kg) o.a. once daily for 4 weeks before LPS administration	CUR lowered IL-1*β*, IL-6, and TNF-*α* and improved liver apoptosis by suppressing phosphatidylinositol 3-kinase/protein kinase B (PI3K/AKT) signaling pathway and inhibiting cyclic AMP-responsive element-binding protein (CREB)/caspase expression and decreased oxidative stress-associated protein-expressing. CUR regulated serum alanine transaminase (ALT), aspartate transaminase (AST), and alkaline phosphatase (ALP), accelerated liver antioxidant enzymes, such as SOD, catalase (CAT), GSH, and glutathione peroxidase (GSH-px) levels, and inhibited activation of the mitogen-activated protein kinases/c-Jun NH2-terminal kinase (P38/JNK) cascade in the livers of LPS-induced rats	CUR exhibited protective effects in LPS-induced mice by inhibiting inflammatory signaling pathway through IKK/NF kappa B pathway suppression and proinflammatory cytokine reduction and limiting PI3K/AKT-related signaling pathway

Kumari et al., 2017 [30]	Mice Swiss albino	Group I: control Group II: LPS Group III: LPS+CUR (20 mg/kg) Group IV: LPS+CUR (10 mg/kg) Group V: LPS+DMSO (vehicle) Group VI: LPS+DEX (dexamethasone -1 mg/kg)	LPS-induced ROS measurement in BALF Nitrite level measurement TNF-a level determination MPO activity in lungs Assessment of capillary leakage Inflammation determination of hydroxyproline (Hyp) content as collagen marker in lungs Histopathological determination of lung fibrosis Survival rate	LPS (10 mg/kg) i.p.	CUR (20 mg/kg, i.p. and 10 mg/kg, i.n.) dissolved in DMSO an hour before LPS administration	CUR ameliorates oxidative damage caused by LPS-induced ROS and nitrite level. LPS-induced TNF-a level was ameliorated by CUR. CUR suppresses neutrophil infiltration and lung inflammation. CUR protects against LPS-induced histopathology and capillary damage. CUR ameliorates LPS-induced collagen deposition in the lungs and liver. CUR ameliorated LPS-induced TGF-*β*1, TLR-4, and iNOS expression in the lungs. CUR protects against LPS- induced lethality	CUR at a lower dose (20 mg/kg, i.p.) can inhibit inflammation, oxidative damage, and fibrotic changes in murine models

Liu et al., 2017 [26]	Rats albino healthy (male)	Group I: control Group II: CLP Group III: CLP+CUR (50 mg/kg) Group IV: CLP+CUR (100 mg/kg)	Measurement of lung W/D ratio Measurement of lung injury in BALF Histopathological examination of the lung Measurement of MPO, MDA, SOD, and catalase enzyme activities Measurement of inflammatory cytokines	CLP	CUR (50 mg/kg or 100 mg/kg) dissolved in saline o.a. The dose was continued for 45 consecutive days	CUR administration significantly reduced CLP-induced inflammation, pulmonary edema, and chronic lung injury (CLI). CUR treatment significantly reduced MPO activity and inflammatory cell accumulation in the BALF, and also protein level, MDA, SOD, and W/D ratio were significantly reduced in the lung tissues. Also, CUR reduced the expression of IL-1b, TNF-a, and MIF levels in the lung tissues	CUR can ameliorate CLP-induced CLI in male albino rats

Maa et al., 2017 [20]	Mice C57BL/6 (male)	Group I: control (carboxymethylcellulose solution) Group II: LPS Group III: LPS+CUR (20 mg/kg)	Histopathological analyses Biochemical levels of serum aspartate transaminase (AST) and blood urea nitrogen (BUN) Detection of F4/80+CD11c+ cells by flow cytometry Western blotting for detection of miR-155	LPS (10 mg/kg) i.p.	CUR (20 mg/kg) in 0.5% carboxymethylcellulose o.a. for 3 days before LPS administration	CUR effectively protected mice from sepsis as evidenced by decreasing histological damage, reducing AST and BUN levels, and the proportion of macrophages in the spleen (31.1% vs. 13.5%). MicroRNA-155 levels and cytokines were also reduced in CUR-treated mice	CUR improves the histopathological and biochemical responses of sepsis and the LPS-induced inflammatory response is mediated by miR-155 expression

Chen et al., 2018 [21]	Mice BALB/c (male)	Group I: sham Group II: CLP Group III: vehicle (CLP+corn oil) Group IV: CLP+CUR (50 mg/kg) Group V: CLP+CUR (100 mg/kg) Group VI: CLP+CUR (200 mg/kg)	Magnetic isolation of CD4+ T and Treg cells Cytokine measurements RT-qPCR Western blotting Flow cytometry Histopathological examination	CLP	CUR (50, 100, 200 mg/kg). Twelve-hour post-CLP was induced, via administration intragastric	CUR significantly alleviated inflammatory injury of the lung and kidney in septic mice and improved survival after CLP. The suppressive function of Treg cells was enhanced and the plasma levels of IL-10 increased after treatment with CUR. TNF-*α* and IL-6 levels were reduced in septic mice treated with CUR	CUR attenuates sepsis-induced acute organ dysfunction by preventing inflammation and enhancing the suppressive function of Tregs

CLP: cecal ligation and puncture; LPS: lipopolysaccharide; i.p.: intraperitoneal; i.n.: intranasal; o.a.: oral administration; EB: Evans blue; BBB: blood-brain barrier; BALF: bronchoalveolar lavage fluid; MPO: myeloperoxidase; SOD: superoxide dismutase; MDA: malondialdehyde; ALI: acute lung injury; ROS: reactive oxygen species; GSH: glutathione; cTnI: cardiac troponin I; EF: ejection fraction; FS: fractional shortening; ALT: alanine transaminase; AST: aspartate transaminase; ALP: alkaline phosphatase; CAT: catalase; GSH-px: glutathione peroxidase; BUN: blood urea nitrogen; CLI: chronic lung injury; iNOS: inducible nitric oxide synthase; cytokines: IL-1b, IL-4, IL-6, IL-10, TNF*α*, IFN-*γ*, MIP-1a, TGF-*β*.

**Table 2:** SYRCLE's risk of bias analysis.

Item	Memis et al. 2008 [28]	Vachharajani et al. 2010 [31]	Xiao et al. 2012 [27]	Savcun et al. 2013 [29]	Xu et al. 2013 [24]	Yang et al. 2013 [25]	Zhao et al. 2016 [22]	Zhong et al. 2016 [23]	Kumari et al. 2017 [30]	Liu et al. 2017 [26]	Maa et al. 2017 [20]	Chen et al. 2018 [21]
1. Was the allocation sequence adequately generated and applied?	U	U	U	U	U	U	U	U	U	U	U	U
2. Were the groups similar at baseline or were they adjusted for confounders in the analysis?	Y	Y	Y	Y	Y	Y	Y	Y	Y	Y	Y	Y
3. Was the allocation adequately concealed?	U	U	U	U	U	U	U	U	U	U	U	U
4. Were the animals randomly housed during the experiment?	Y	U	Y	Y	Y	U	Y	Y	Y	U	Y	Y
5. Were the caregivers and/or investigators blinded from knowledge which intervention each animal received during the experiment?	U	U	U	U	U	U	U	U	U	U	U	U
6. Were animals selected at random for outcome assessment?	U	U	U	U	U	U	U	U	U	U	U	U
7. Was the outcome assessor blinded?	U	U	U	U	U	U	U	U	U	U	U	U
8. Were incomplete outcome data adequately addressed?	U	U	U	U	U	U	U	U	U	U	U	U
9. Are reports of the study free of selective outcome reporting?	U	U	U	U	U	U	U	U	U	U	U	U
10. Was the study apparently free of other problems that could result in high risk of bias?	U	U	U	U	U	U	U	U	U	U	U	U

Y: yes; N: no; U: unclear.

## Data Availability

The authors confirm that data supporting the findings of this study are available in the article.

## Supplementary Materials

Supplementary Materials As supplementary material, we present the 27-item PRISMA 2020 checklist, used for the design and reporting of a robust protocol for this systematic review.
